# Prognostic Biomarker Identification Through Integrating the Gene Signatures of Hepatocellular Carcinoma Properties

**DOI:** 10.1016/j.ebiom.2017.04.014

**Published:** 2017-04-12

**Authors:** Jialin Cai, Bin Li, Yan Zhu, Xuqian Fang, Mingyu Zhu, Mingjie Wang, Shupeng Liu, Xiaoqing Jiang, Jianming Zheng, XinXin Zhang, Peizhan Chen

**Affiliations:** a Translational Medicine Research Center, Ruijin Hospital North, Shanghai Jiao Tong University School of Medicine, Shanghai 201821, PR China; b Biliary Tract Surgery Department I, Eastern Hepatobiliary Surgery Hospital, Secondary Military Medical University, Shanghai 200433, PR China; c Diagnosis and Treatment Center of Malignant Biliary Tract Diseases, Secondary Military Medical University, Shanghai 200433, PR China; d Department of Pathology, Changhai Hospital, Secondary Military Medical University, Shanghai 200433, PR China; e Department of Infectious Diseases, Ruijin Hospital, School of Medicine, Shanghai Jiao Tong University, Shanghai 200025, PR China

**Keywords:** Hepatocellular carcinoma, Overall survival, Biomarker, Network, Gene signature

## Abstract

Many molecular classification and prognostic gene signatures for hepatocellular carcinoma (HCC) patients have been established based on genome-wide gene expression profiling; however, their generalizability is unclear. Herein, we systematically assessed the prognostic effects of these gene signatures and identified valuable prognostic biomarkers by integrating these gene signatures. With two independent HCC datasets (GSE14520, N = 242 and GSE54236, N = 78), 30 published gene signatures were evaluated, and 11 were significantly associated with the overall survival (OS) of postoperative HCC patients in both datasets. The random survival forest models suggested that the gene signatures were superior to clinical characteristics for predicting the prognosis of the patients. Based on the 11 gene signatures, a functional protein-protein interaction (PPI) network with 1406 nodes and 10,135 edges was established. With tissue microarrays of HCC patients (N = 60), we determined the prognostic values of the core genes in the network and found that RAD21, CDK1, and HDAC2 expression levels were negatively associated with OS for HCC patients. The multivariate Cox regression analyses suggested that CDK1 was an independent prognostic factor, which was validated in an independent case cohort (N = 78). In cellular models, inhibition of CDK1 by siRNA or a specific inhibitor, RO-3306, reduced cellular proliferation and viability for HCC cells. These results suggest that the prognostic predictive capacities of these gene signatures are reproducible and that CDK1 is a potential prognostic biomarker or therapeutic target for HCC patients.

## Highlights

• Eleven gene signatures have promising values for the OS prediction of HCC patients who undergo the surgical treatments.
• Prediction results for the 11 gene signatures were in moderate concordance, and genes included were functionally linked.
• CDK1 is an independent prognostic biomarker and a potential therapeutic target for HCC patients.

Various gene signatures for hepatocellular carcinoma (HCC) patients have been reported; however, their generalizability is unclear. Using two HCC patient datasets, we assessed the prognostic values of these gene signatures and identified 11 gene signatures that were associated with overall survival for postoperative HCC patients in both cohorts. Genes derived from these signatures formed a functional protein-protein interaction network with 1,406 nodes and 10,135 edges, and the expression levels of three core genes, RAD21, CDK1, and HDAC2, in the network were negatively associated with the overall survival of HCC patients. Further studies suggested that CDK1 is an independent prognostic factor and that it is a potential therapeutic target for HCC patients.

## 1 Introduction

For men and women worldwide, liver cancer ranks as the second and sixth leading cause of cancer deaths, respectively (Torre et al., 2015). In 2012, there were an estimated 782,500 new cases of liver cancer and 745,500 deaths worldwide, and the incidence of the disease is rising (Torre et al., 2015). Hepatocellular carcinoma (HCC) account for > 90% of primary liver cancer cases, and epidemiological studies have revealed that chronic hepatitis B virus (HBV) or hepatitis C virus (HCV) infection, exposure to aflatoxin, alcohol consumption, cigarette smoking, diabetes, and susceptibility genetic factors are major risk factors for HCC (Donato et al., 2002, El-Serag, 2012, Yang et al., 2011). The prognosis for HCC patients is poor: the 5-year survival rate for localized HCC patients is 30.5%, and this rate drops below 5% for those with distant metastases according to the Surveillance, Epidemiology, and End Results (SEER) database (El-Fattah et al., 2017, Oweira et al., 2017). For patients at early disease stages, liver resection is the most effective treatment methods; however, fewer than 30% of HCC patients are eligible for this treatment, and approximately 70% of them will relapse within 5 years of treatment (Intaraprasong et al., 2016). Thus, it is necessary to identify those prognostic factors and systematically evaluate patient characteristics to guide the postoperative treatments and surveillance, which may improve the prognosis of HCC patients.

Similar to other solid tumors, the characteristics such as tumor size, tumor differentiation, tumor node numbers, vascular invasion, and metastasis status are important prognosis factors for HCC patients (Noh et al., 2016). These characteristics constitute the tumor-node-metastasis (TNM) classification system for HCC patients (Sobin, 2003). In addition to these tumor characteristics, biomarkers for preserved liver function and the liver damage status of the HCC patients, such as the Child-Pugh stage; the α-fetoprotein (AFP), bilirubin, and albumin levels; and ECOG status are also associated with the prognosis of HCC patients. These additionally markers have led to the establishment of various conventional staging systems, including the Japan Integrated Staging (JIS) system (Kudo et al., 2003), the Barcelona Clinic Liver Cancer (BCLC) classification system (Llovet et al., 1999), the Cancer of the Liver Italian Program (CLIP) scoring system (No-author-listed, 1998) and the Chinese University Prognostic Index (CUPI) scoring system (Leung et al., 2002). These systems are widely used to guide the treatment methods and/or predict the outcomes of HCC patients. However, the clinical performance of these systems depends on the patient characteristics, the treatments performed, and the disease etiology of the patients (Marrero et al., 2010, Subramaniam et al., 2013). Moreover, although these staging and scoring systems can stratify the HCC patients into appropriate risk categories, a great deal of divergence remains within each risk category due to the molecular heterogeneity of tumor cells and the tumor microenvironment (Fridman et al., 2012). An in-depth characterization and understanding of the molecular basis of the tumor and its corresponding microenvironment are critical for improving the diagnosis, identifying prognostic and predictive biomarkers, and developing effective therapeutic strategies (Koren and Bentires-Alj, 2015).

Genome-wide expression profiling methods provide detailed information regarding the diversity of diseases and are valuable for the disease diagnosis, therapeutic response prediction and prognosis evaluation. Currently, many studies have assessed the prognostic effects of array-based gene expression signatures obtained from HCC tumors (Andersen et al., 2010, Boyault et al., 2007, Cairo et al., 2008, Chew et al., 2012, Chiang et al., 2008, Coulouarn et al., 2008, Hoshida et al., 2009, Iizuka et al., 2003, Kaposi-Novak et al., 2006, Kim et al., 2012, Ko et al., 2014, Kurokawa et al., 2004, Lee et al., 2004, Lim et al., 2013, Minguez et al., 2011, Roessler et al., 2010, Roessler et al., 2012, Sakai et al., 2008, Villanueva et al., 2008, Wang et al., 2007, Woo et al., 2010, Woo et al., 2008, Yamashita et al., 2008, Ye et al., 2003, Yoshioka et al., 2009) or from adjacent, non-tumor tissues (Budhu et al., 2006, Hoshida et al., 2008, Okamoto et al., 2006). These investigations have identified gene signatures that predict recurrence and/or mortality for HCC patients; however, none has entered clinical use, perhaps due to their low reproducibility and lack of standardized determination methods. Interestingly, overlapping genes between these gene signatures are rare, and this rarity may be related to the disease stage of the patients, the main hypothesis of the study, the platform applied, and/or the data mining methods that were utilized. However, these gene signatures with little overlap might be functionally linked with each other and form a systematic molecular regulation network that is robust for patient stratification. Herein, we systematically evaluated the generalization of the prognostic gene signatures in independent HCC case cohorts and established a functional protein-protein interaction (PPI) network for gene signatures that have reproducible prognostic values. Through the PPI network topological analysis, we identified those critical molecules in the network and determined their values as biomarkers for prognosis assessment or as therapeutic targets for HCC patients.

## 2 Methods and Materials

### 2.1 Identification of Candidate HCC Prognostic Gene Signatures

The PubMed database, published up to April 30, 2016, was searched to identify gene signatures that have prognostic effects for HCC patients. The terms (“liver cancer” or “hepatocellular carcinoma”) AND (“prognosis” or “overall survival” or “outcome” or “mortality”) AND (“gene expression” or “gene signature” or “expression profiling”) were used. A total of 1713 original publications were identified in our initial database search. Through checking the titles and abstracts of studies, we excluded those studies that were only performed in the cellular or animal models but not in the human populations. Comments, reviews, abstracts or short communications without sufficient information were also excluded. Thus, 89 publications were selected and further checked in full length to identify gene signatures that might be associated with the overall survival (OS) of HCC patients. Excluded were gene signatures derived from cell lines, specific biological signaling, microRNA expression, long non-coding RNA expression, and methods based on real-time PCR (RT-PCR). Gene signatures derived from tumor microenvironment samples were also excluded because we specifically focused on gene expression information derived from the tumor tissues here. A total of 30 gene signatures from 25 studies were identified, and their prognostic performance in HCC patients was determined [15-39] (Table 1).

**Table 1:** Gene signatures included in the nearest-template prediction studies of the GSE14520 (N = 242) and GSE54236 (N = 78) datasets.

Study	Year	Signature names (Molecular Signature Database [MSIGBD]^a^)	Signature name in the study	No. of genes in the signature	Genes covered in GSE14520	Samples with the signature (GSE14520)^b^	Genes covered in GSE54236	Samples with the signature (GSE54236)^b^
Iizuka et al.	2003	IIZUKA_LIVER_CANCER_EARLY_RECURRENCE_DN	Recurrence_Lizuka	12	12 (100.0%)	NA	12 (100.0%)	NA
Ye et al.	2003	YE_METASTATIC_LIVER_CANCER	Metastasis_Ye	28	28 (100.0%)	NA	27 (96.4%)	NA
Lee et al.	2004	LEE_LIVER_CANCER_POOR-SURVIVAL_UP, _DN	Lee_OS	360	316 (87.8%)	202 (83.5%)	355 (98.6%)	69 (88.5%)
Korukawa et al.	2004	Early_recurrence_signature^c^	Recurrence_Korukawa	19	17 (89.5%)	NA	19 (100.0%)	NA
Kaposi-Novak et al.	2006	NOVAK_LIVER_CANCER_MET_UP, _DN	MET_Kaposi-Novak	24	24 (100.0%)	66 (27.3%)	24 (100%)	24 (30.8%)
Boyault et al.	2007	BOYAULT_LIVER_CANCER_SUBCLASS_G3_UP, _DN	G3_Boyault	239	239 (100.0%)	192 (79.3%)	239 (100.0%)	64 (82.1%)
Boyault et al.	2007	BOYAULT_LIVER_CANCER_SUBCLASS_G56_UP,_DN	G5/6_Boyault	29	29 (100.0%)	78 (32.2%)	29 (100.0%)	21 (26.9%)
Boyault et al.	2007	BOYAULT_LIVER_CANCER_SUBCLASS_G6_UP,_DN	G6_Boyault	84	84 (100.0%)	69 (28.5%)	84 (100.0%)	26 (33.3%)
Wang et al.	2007	WANG_RECURRENT_LIVER_CANCER_UP,_DN	Recurrence_Wang	36	32 (88.9%)	75 (31.0%)	36 (100.0%)	20 (25.6%)
Cairo et al.	2008	C2_POOR-PROGNOSIS_UP, _DN	C2_Cario	16	16 (100.0%)	102 (42.1%)	16 (100.0%)	29 (37.2%)
Chiang et al.	2008	CHIANG_LIVER_CANCER_SUBCLASS_CTNNB1_UP,_DN	CTNNB1_Chiang	346	280 (80.9%)	134 (55.4%)	343 (99.1%)	57 (73.1%)
Chiang et al.	2008	CHIANG_LIVER_CANCER_SUBCLASS_INTERFERON_UP,_DN	Interfron_Chiang	78	57 (73.1%)	85 (35.15)	77 (98.7%)	33 (42.3%)
Chiang et al.	2008	CHIANG_LIVER_CANCER_SUBCLASS_PROLIFERATION_UP,_DN	Proliferation_Chiang	357	299 (83.8%)	198 (81.8%)	353 (98.9%)	68 (87.2%)
Coulouarn et al.	2008	COULOUARN_LIVER_CANCER_TGF_BETA_LATE_VS_EARLY_UP, _DN	TGFB_Coulouarn	249	215 (86.3%)	110 (45.5%)	244 (98.0%)	42 (53.8%)
Sakai et al.	2008	SAKAI_TUMOR_INFILTRATING_MONOCYTES_UP,_DN	Monocyte_Sakai	108	104 (96.3%)	51 (21.1%)	106 (98.1%)	23 (29.5%)
Woo et al.	2008	WOO_LIVER_CANCER_RECURRENCE_UP,_DN	Recurrence_Woo	185	185 (100.0%)	176 (72.7%)	185 (100.0%)	61 (78.2%)
Yamashita et al.	2008	YAMASHITA_LIVER_CANCER_STEM_CELL_UP,_DN	CSC_Yamashita	112	104 (92.9%)	143 (59.1%)	112 (100.0%)	42 (53.8%)
Yamashita et al.	2008	YAMASHITA_LIVER_CANCER_WITH_EPCAM_UP,_DN	EPCAM_Yamashita	70	65 (92.9%)	135 (55.8%)	68 (97.1%)	32 (41.0%)
Hoshida et al.	2009	HOSHIDA_LIVER_CANCER_SUBCLASS_S3,_S1	S1_Hoshida	498	492 (98.8%)	170 (70.2%)	498 (100.0%)	60 (76.9%)
Hoshida et al.	2009	HOSHIDA_LIVER_CANCER_SUBCLASS_S3,_S2	S2_Hoshida	379	370 (97.6%)	179 (74.0%)	379 (100.0%)	53 (67.9%)
Yoshioka et al.	2009	YOSHIOKA_LIVER_CANCER_EARLY_RECURRENCE_UP,_DN	Recurrence_Yoshioka	105	80 (76.2%)	NA	100 (95.2%)	NA
Andersen et al.	2010	ANDERSEN_LIVER_CANCER_KRT19_UP, _DN	CK19_Andersen	110	96 (87.3%)	177 (73.1%)	109 (99.1%)	60 (76.9%)
Roessler et al.	2010	ROESSLER_LIVER_CANCER_METASTASIS_UP,_DN	Metastasis_Roessler	161	148 (91.9%)	NA	155 (96.3%)	NA
Woo et al.	2010	WOO_LIVER_CANCER_CHOLANGIOCA_LIKE_UP, _DN	CC_Woo	625	599 (95.8%)	205 (84.7%)	616 (98.6%)	67 (85.9%)
Minguez et al.	2011	MINGUIZ_LIVER_CANCER_VASCULAR_INVASION_UP, _DN	VI_Minguez	35	33 (94.3%)	109 (45.0%)	34 (97.1%)	31 (39.7%)
Chew et al.	2012	Lymphocyte_infiltration_signature^c^	Lymphocyte_Chew	14	14 (100.0%)	NA	13 (92.9%)	24 (30.8%)
Kim et al.	2012	Overall_survival_signature^c^	OS_Kim	65	65 (100.0%)	174 (71.9%)	65 (100.0%)	59 (75.6%)
Roessler et al.	2012	Poor_outcome_signature^c^	G2_Roessler	10	9 (90.0%)	35 (14.5%)	10 (100.0%)	NA
Lim et al.	2013	Disease_free_survival_signature^c^	DFS_Lim	30	18 (60.0%)	NA	26 (86.7%)	NA
Ko et al.	2014	VDAC1_signature^c^	VAG_Ko	45	45 (100.0%)	74 (30.6%)	45 (100.0%)	19 (24.4%)

a MSIGDB: www.broadinstitute.org/gsea/msigdb.

b Samples enriched with good or poor prognosis according to the nearest-template prediction (NTP) method for each gene signature (FDR < 0.05). NA means the NTP method failed to classify any samples for the gene signature.

c Gene signature not included in the MSIGDB database and a brief introduction was provided.

### 2.2 Identification of Gene Expression Datasets and Data Processing

The NCBI GEO (http://www.ncbi.nlm.nih.gov/geo/) and ArrayExpress (http://www.ebi.ac.uk/arrayexpress/) databases were searched to identify datasets that had determined the prognostic effects of genome-wide gene expression levels. Eligible datasets should have determined the gene expression level in the HCC tumor tissues at the genome-wide level and provided the OS time and the corresponding status (alive or dead) at the last follow-up. OS was defined as the time range from the day of surgery to death or the last follow-up. Datasets that only provided gene expression levels in adjacent normal tissues or did not provide prognostic information for the patients were excluded. Initially, 97 datasets were identified for HCC, and 78 were excluded because they were based on formalin-fixed, paraffin-embedded (FFPE) tissues, cell lines, non-tumor tissues, or animal models. Additionally, 17 datasets that did not provide prognostic information for the HCC patients were also excluded after we carefully checked the original reports or emailed the authors. GEO: GSE14520 (Roessler et al., 2010, Roessler et al., 2012) and GEO: GSE54236 (Villa et al., 2016) datasets fully met the inclusion criteria and were defined as the validation case-cohorts for the gene signatures in our current study.

GSE14520 dataset consisted of tissues from 242 patients with primary HCCs, who underwent radical resections between 2002 and 2003 at the Liver Cancer Institute and Zhongshan Hospital (Fudan University, Shanghai, China). From this cohort, 220 samples were analyzed with the Affymetrix HT Human Genome U133A Array (GPL3921 platform with 22,277 probes), and 22 samples were analyzed with the Affymetrix Human Genome U133A 2.0 Array (GPL571 platform with 22,277 probes). The raw expression data were processed and normalized by the Robust Multi-Array Average (RMA) method and global median centering. The array series was combined according to the probe IDs, and batch effects were adjusted with empirical Bayes methods in the Combat package of R software. For genes with more than one probe set, the mean level was calculated to obtain the individual gene expression level. Detailed clinical information was provided by Roessler et al. (Roessler et al., 2010).

GSE54236 dataset consisted of tissues from 78 primary HCC patients who received surgery at the Modena Gastroenterology Unit, Italy (Villa et al., 2016). The gene expression levels of the HCC tissues were determined with the Agilent Whole Human Genome Microarray 4 × 44K array with 41,000 probes. The expression levels were processed with Agilent Feature Extraction Software, and the quantile normalized log2 signal intensity data were downloaded from the GEO datasets. The mean level for probes from the same gene was calculated to determine the gene expression level. Detailed information for the patients was provided by Villa et al. (Villa et al., 2016).

### 2.3 Recruitment of HCC Patients

A retrospective study was performed for HCC patients (N = 60) who received surgery treatment with curative intent from July 2012 to February 2014 at the Eastern Hepatobiliary Surgery Hospital of the Second Military Medical University. Patients were included if they received curative resection surgery for the first time without any previous anticancer treatment including chemotherapy or radiotherapy. Patients were excluded if they received palliative treatments, had a distant metastasis, had a history of other malignancies, received a liver transplant or were unwilling to participate in the study. All patients were diagnosed with HCC based on the pathological examination. Personal characteristics and clinicopathological characteristics of the patients were extracted from the medical records and assessed by the clinicians. The results of the preoperative biochemical tests and image evaluations were also retrieved. The resection treatments and surgical procedures were performed following the general guidelines that had been designed according to tumor size, location, and liver functional reserve (Wang et al., 2010a). Patient follow-up was performed by telephone calls or checking the medical records at half-year intervals, and the last follow-up was performed in September 2016.

An independent validation case cohort was recruited from November 2009 to March 2010 in the same hospital (N = 78). The inclusion and exclusion criteria were similar to the previous case cohort. The last follow-up was performed September 2012 with the longest follow-up period extending up to 39 months. Detailed participant information is provided as Supplementary Table 3. Each participant provided written consent, and the institutional review board of the Second Military Medical University approved the study.

### 2.4 Immunohistochemical (IHC) Staining Methods

For the HCC patients, FFPE tumor tissues were used by the National Engineering Center for Biochip, Shanghai, to construct tissue microarrays (TMAs). For each patient, a 0.75-mm diameter core of the cancer FFPE tissue was punched and arranged in the TMA blocks. Sections of the TMAs (6-μm thick) were used to determine the expression levels of RAD21, CDK1, and HDAC2 following general IHC staining protocols. In brief, the TMAs were deparaffinized with xylene and rehydrated with graded ethanol solutions. To quench endogenous peroxidase activity, the TMA was treated with 3% hydrogen peroxide for 15 min at room temperature. Then, the sections were put into citric acid solution (pH = 6.0) and boiled at 95 °C for 40 min. After cooling, the sections were washed three times with phosphate-buffered saline (PBS) and blocked in 5% fetal bovine serum for 15 min at room temperature. Primary antibodies for CDK1 (1:150; Abcam, USA), HDAC2 (1:200; Abcam), and RAD21 (1:50; Santa Cruz, USA) were added to the sections, which were incubated at 4 °C overnight. After washing three times with PBS, the sections were incubated with a peroxidase-polymer labeled rabbit anti-mouse secondary antibody, followed with diaminobenzidine staining to detect peroxidase activity. Finally, the sections were counterstained with hematoxylin and differentiated in hydrochloric acid alcohol, followed by dehydration and mounting.

Expression was assessed by the staining intensity and the distribution of the positive cells. Intensity was categorized as 0–3 (0, negative; 1, weak; 2, moderate; 3, strong), and the distribution of positive cells was grouped as 1, 0–25%; 2, 25–50%; 3, 50–75%; and 4, > 75%. The immunoreactive score (IRS) was calculated by multiplying the intensity with the distribution of positive cells (Wang et al., 2012), and the median IRS score was applied to categorize the HCC patients into high and low protein expression levels.

### 2.5 Construction of the PPI Network

For those gene signatures significantly that were associated with the prognosis of the HCC patients in both case cohorts, PPI networks for these genes were constructed with the Reactome FI plugin of the Cytoscape software (Version 3.2.0; http://www.cytoscape.org/) (Shannon et al., 2003, Wu et al., 2010). With the Network Analyzer plugin (Version 1.0; http://med.bioinf.mpi-inf.mpg.de/netanalyzer/) (Assenov et al., 2008), the basic topological parameters, including the degree of node distribution, the shortest path length distribution, and the average clustering coefficient distribution were assessed.

### 2.6 Cell Culture and siRNA Transfection Methods

The human liver cancer cell lines Hep3B, SMMC-771 and Huh-7 were purchased from the Cell Bank of Chinese Academy of Sciences (Shanghai, China). The CSQT-2 cell line, which was derived from the portal vein tumor thrombi (PVTT) of an HCC patient and established by Dr. Cheng's lab (Wang et al., 2010b), was kindly provided by Dr. Cheng's lab. All cell lines were cultured in Dulbecco's modified Eagle medium supplemented with 10% fetal bovine serum, 100 μg/mL of streptomycin, 4.5 mg/mL of d-glucose, 300 mg/L of l-glutamine, and 110 mg/L of sodium pyruvate. The cell lines were maintained in an incubator with a humidified atmosphere of 5% CO_2_ at 37 °C.

In the siRNA transfection assays, 5 × 10^5^ cells/well were plated in 6-well cell culture plates. The siRNA duplexes for CDK1, RAD21, and HDAC2 or the scramble control were synthesized by GenePharma (Shanghai, China) and were transfected with Lipofectamine 2000 reagent (Life Technologies, USA) at a concentration of 40 nmol/L following the manufacturer's guidelines. After 48 h, the endogenous protein levels in the transfected cells were determined by Western blotting.

### 2.7 Determination of Cell Viability and Proliferation

Cells (2000 cells/well) were plated in 96-well plates, and 24 h later, the siRNA duplexes for CDK1, RAD21, and HDAC2 or the scramble control were transfected into CSQT-2 cells (40 nmol/L). 24, 48 and 72 h after transfection, the CCK-8 solution was added to the cells, and the preparations were incubated for another 4 h before the absorption values (450 nm) were determined.

To determine the anti-cancer activities of the CDK1 selective inhibitor RO-3306 (Selleck, China), liver cancer cells (3000 per well) were plated in 96-well plates. After 24 h, RO-3306 was added to the cells at concentrations of 0 (vehicle, DMSO), 0.78, 1.56, 3.13, 6.25, 12.5, or 25 μM. The cells were cultured for another 48 h, and CCK-8 solution was applied to determine the cellular viability following the manufacturer's guidelines. To determine the anti-proliferation activity of RO-3306, liver cancer cells (3000 per well) were plated in 96-well plates, and the vehicle (DMSO) or RO-3306 (12.5 μM) was added; after 24, 48, or 72 h, CCK-8 was applied to determine the cellular proliferation.

### 2.8 Western Blotting Methods

Cells were lysed with radio-immunoprecipitation assay (RIPA) buffer containing a cocktail of protease inhibitors (Sigma, USA). Protein concentrations were measured by the bicinchoninic acid assay (Sigma, USA), and total protein (30 μg) was separated on 10% SDS-PAGE gels and transferred onto polyvinylidene fluoride (PVDF) membranes (Millipore, USA). Membranes were blocked with 5% skim milk for 1 h and then probed overnight at 4 °C with the same antibodies as in the IHC staining tests. After washing 3 times, the PVDF membranes were incubated with secondary horseradish peroxidase-linked secondary antibody for 1 h at room temperature. The signaling intensity was determined with an enhanced chemiluminescence detection reagent and visualized with X-ray films.

### 2.9 Statistical Analyses

To determine the prognostic effects of the gene signatures, the nearest template prediction (NTP) method implemented in the Gene Pattern software (Broad Institute of Harvard and MIT, Boston, MA) was used to make class prediction of the HCC patients based on the gene-expression data and the list of signature genes (Hoshida, 2010). Templates of the “poor prognosis” and “good prognosis” patterns for each gene signature were defined as those genes associated with worse or better outcomes, respectively. Subjects closer to “poor” or “good” prognosis templates with a false discovery rate (FDR) < 0.05 were classified as poor or good outcome, respectively. Those with the FDR > 0.05 were recognized as an unclassified group. Cramer's V test was calculated to test the concordance rate of the prediction class for the patients by the gene signatures (Fan et al., 2006). Values < 0.40 indicated a weak correlation between the gene signatures, values between 0.40 and 0.60 indicated substantial correlation, and values > 0.60 indicated strong correlation. Kaplan-Meier plots, together with the log-rank tests, were applied to identify gene signatures that were associated with the OS of the patients. Univariate Cox regressions were applied to determine the associations between the basic characteristics or the gene signatures and OS of the patients. Due to the collinearity for the variables of the characteristics and the NTP prediction outcomes, the associations between the variables and OS of patients were assessed with the random survival forest method, which was developed for right-censored data (Ishwaran and Kogalur, 2010). Variable predictiveness was assessed with the variable importance (VIMP) measures for individual factors in the random survival forests model (Ishwaran and Kogalur, 2010). A positive VIMP value indicated that the misspecification detracted from the predictive accuracy in the forest for the variables and that the predictive power of the forest depended on these variables. Zero or negative VIMP values suggested that the variables contributed nothing to predictive accuracy or even contributed noise to the prediction models and that these variables should be filtered out from the models. In the random survival forest, 5000 trees were grown using a log-rank, score-splitting algorithm. For evaluations, the VIMP for each variable was recorded. The analysis was independently repeated 100 times, and the VIMP was averaged for each variable.

For the TMA cohorts from the Eastern Hepatobiliary Surgery Hospital, univariate Cox regressions were performed to evaluate the basic characteristics and the IHC scores of the biomarkers that were associated with OS. Backward stepwise multivariate Cox regression analyses were performed to identify the independent factors that contribute to the OS of HCC patients. Only significant factors in the univariate analyses were introduced into the multivariate Cox model.

Differences in demographic characteristics and selected variables were evaluated with the χ^2^ test (for categorical variables) or Student's *t*-test (for continuous variables). Comparisons between the multiple groups were performed using one-way ANOVA to determine the overall significance. The data are presented as the means ± SEM, unless otherwise indicated. All analyses were performed with R software (Version 3.3.1; www.r-project.org) and related packages, and P < 0.05 for two-sided tests was recognized as statistically significant.

## 3 Results

### 3.1 The Nearest Template Prediction Results for HCC Patients

**Fig. 1:**
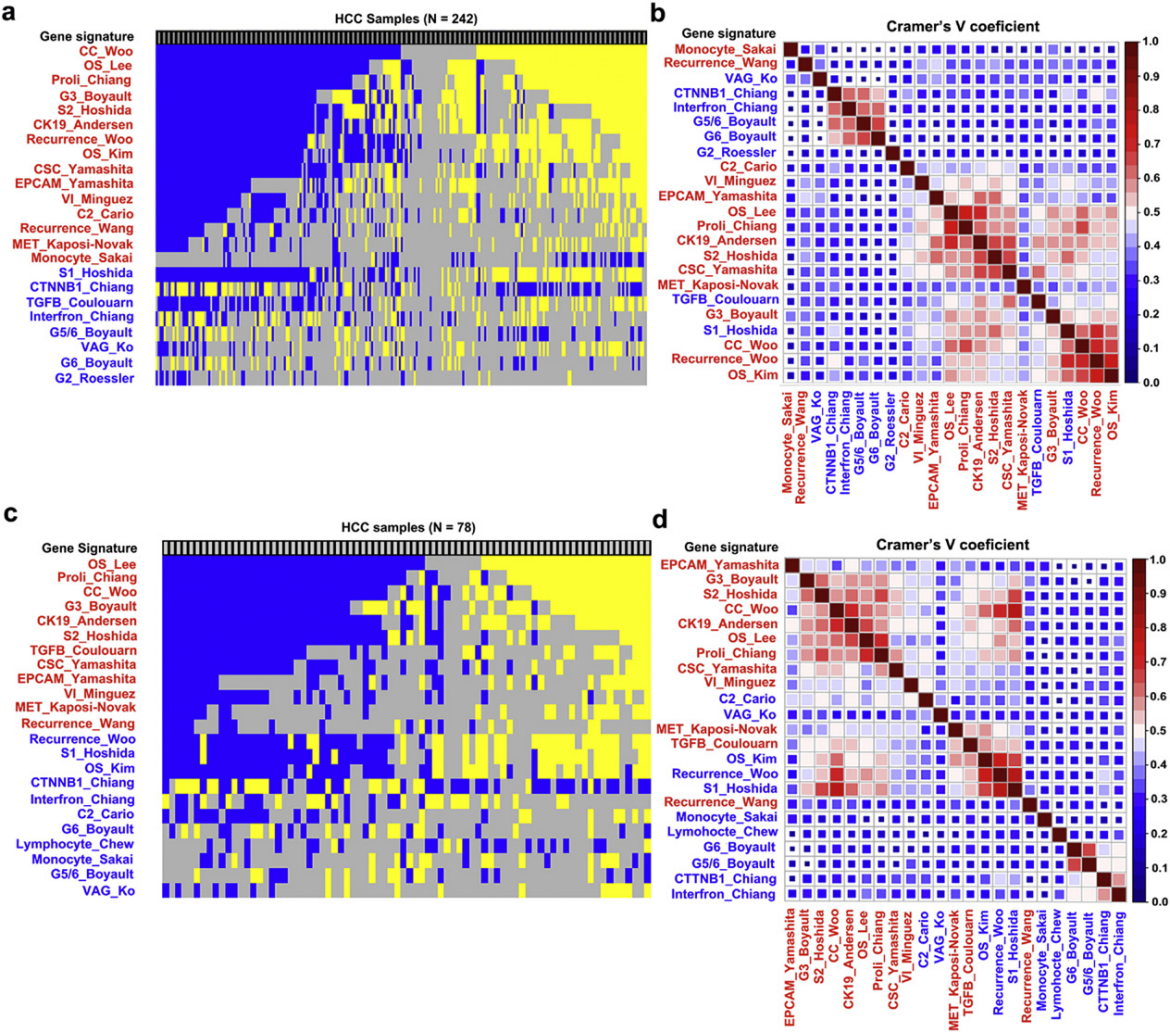
Nearest template prediction (NTP) results and their concordance with gene signatures in the GSE14520 and GSE54236 datasets. (a) NTP results in dataset GSE14520 (N = 242), with each column representing the prediction results for individual patients. Gene signatures suggesting poorer overall survival (OS) are labeled in blue, and signatures that suggest better OS are labeled in yellow (FDR, P < 0.05). The gray column indicates the presence of an unclassified group of patients (FDR, P > 0.05). The left label indicates the gene signature name, as listed in Table 1. (b) Heat map of Cramer's V coefficient values for pair-wise gene signatures in GSE14520; the signatures are clustered according to their degree of correlation. (c) NTP results in dataset GSE54236 (N = 78), with each column representing the prediction result for individual patients. Poorer (FDR, P < 0.05), better (FDR, P < 0.05), or unclassified (FDR > 0.05) outcomes for each patient are labeled in blue, yellow, or gray, respectively. The left label indicates the gene signature name as listed in Table 1. (d) Heat map of Cramer's V coefficient values for pair-wise gene signatures in GSE54236; the signatures are clustered according to their degree of correlation. Gene signatures that are significantly associated with the OS of HCC patients are labeled in red, and those not associated with OS are labeled in blue.

A total of 30 gene signatures were identified that might be associated with the prognosis for HCC patients (Table 1). The number of genes ranged from 10 to 625, and 60% to 100% of these genes were covered by platforms GSE14520 and GSE54236 (Table 1). For each dataset, 23 of the 30 gene signatures successfully classified the HCC patients into poor or good outcomes (FDR P < 0.05). The number of patients enriched with the gene signature ranged from 35 (14.5%) to 205 (84.7%) in GSE14520 and from 19 (24.4%) to 69 (88.5%) in GSE54236 (Table 1). The NTP prediction results are provided in Fig. 1. Cramer's V test suggested that there were three major groups for the prediction results of the gene signatures in the two cohorts (Fig. 1b and d). The first class contained mainly gene signatures that indicated the progenitor tumor cell origin (CSC_Yamashita (Yamashita et al., 2008), EPCAM_Yamashita (Yamashita et al., 2008), CK19_Andersen (Andersen et al., 2010), S2_Hoshida (Hoshida et al., 2009) and C2_Cario (Cairo et al., 2008)); cellular proliferation (Proli_Chiang (Chiang et al., 2008)); and vascular invasion (VI_Minguez (Minguez et al., 2011)). The second class contained those gene signatures of TGF-beta_Coulouarn (Coulouarn et al., 2008), MET_Kaposi-Novak (Kaposi-Novak et al., 2006), G3_Boyault (Boyault et al., 2007), S1_Hoshida (TGFβ-WNT) (Hoshida et al., 2009), Recurrence_Woo (Woo et al., 2008), and OS_Kim (Kim et al., 2012). The third class contained the gene signatures of CTTNB1_Chiang (Chiang et al., 2008), Interferon_Chiang (Chiang et al., 2008), G6_Boyault (CTTNB1_WNT activation) (Boyault et al., 2007), and G5/6_Boyault (CTTNB1_WNT activation) (Boyault et al., 2007).

### 3.2 Association of Gene Signatures With the OS of HCC Patients

**Fig. 2:**
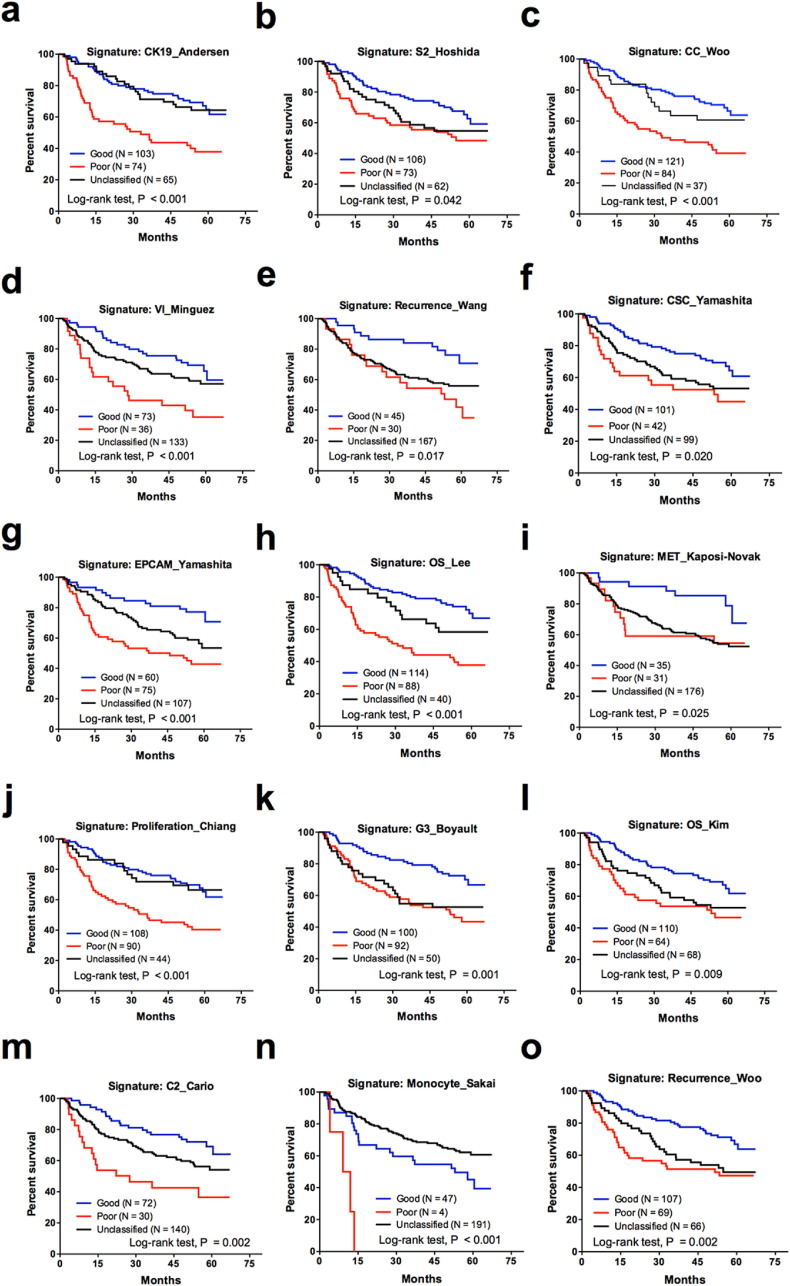
Kaplan-Meier plots and log-rank tests for the 15 gene signatures that are associated with the overall survival of HCC patients in GSE14520.

**Fig. 3:**
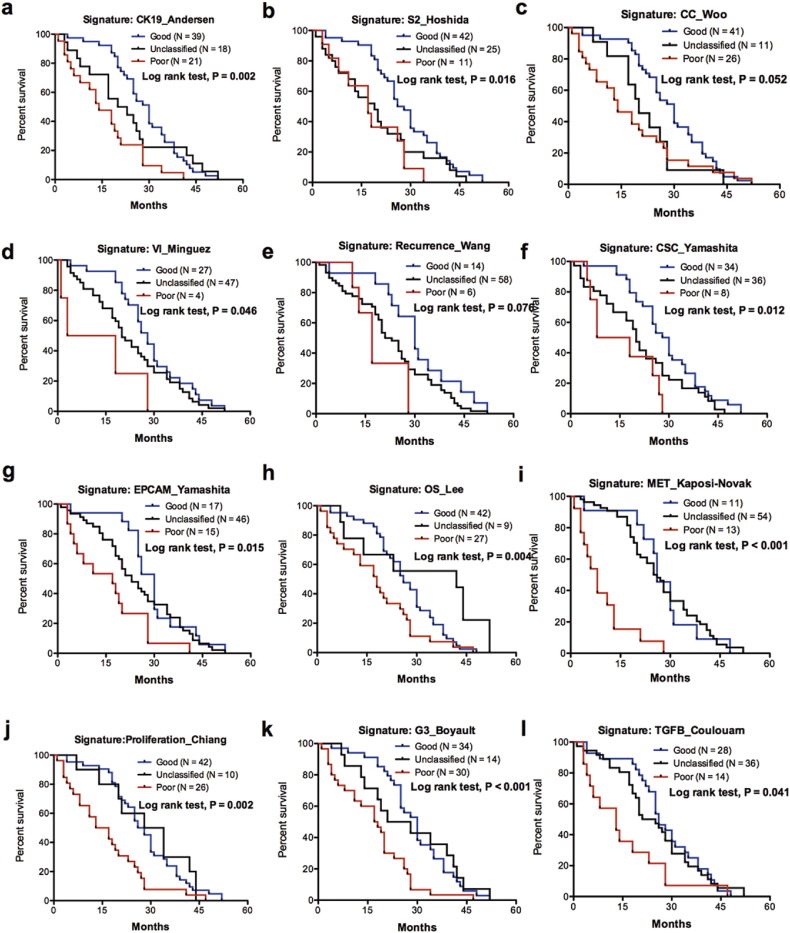
Kaplan-Meier plots and log-rank tests for the 12 gene signatures that are associated with the overall survival of HCC patients in GSE54236.

In dataset GSE14520, the univariate Cox analyses showed that tumor size, nodular number (multiple vs. single), cirrhosis, AFP level, BCLC stage, CLIP stage and TNM stage were associated with the OS of the patients (Table S1). Of the 23 gene signatures in GSE14520, 15 were associated with the OS of the HCC patients, as indicated by Kaplan-Meier plots and log-rank tests (Fig. 1, Fig. 2). For GSE54236, the univariate Cox analyses indicated that the doubling time for HCC was associated with the OS of HCC patients (Table S1). Kaplan-Meier plots showed that, in dataset GSE54236, 12 gene signatures were associated with the OS of the HCC patients (Fig. 1, Fig. 3). Univariate Cox analyses for the gene signatures that categorized HCC groups (poor or unclassified vs. good) are provided as Table S2. Eight and 11 gene signatures were not associated with the OS of HCC patients in GSE14520 (Fig. S1) and GSE54236 (Fig. S2), respectively. Overall, 11 gene signatures were associated with the OS for HCC patients in both case cohorts (Table S3).

**Fig. 4:**
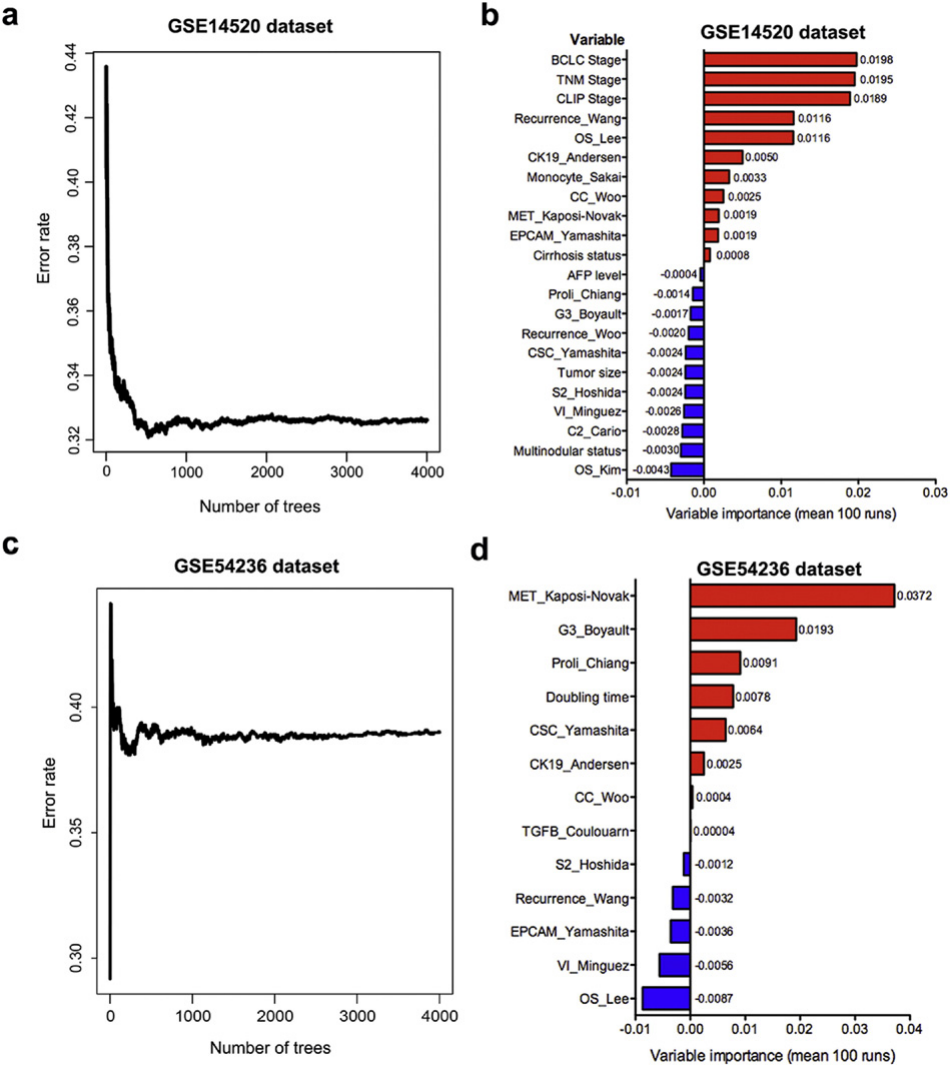
Random survival forests and the corresponding VIMP values for gene signatures in prediction of overall survival for HCC patients in datasets GSE14520 (N = 242) and GSE54236 (N = 78). The error rates according to the number of trees generated in the random survival forest analyses in GSE14520 (a) and GSE54236 and (c). The mean VIMP values for each variable after 100 runs are provided in GSE14520 (b) and GSE54236 (d).

To test the prognostic performance of the gene signatures, a random survival forest algorithm was applied to determine the VIMP values of the basic characteristics and the gene signatures (Fig. 4). In GSE14520, the top-ranked variables after 100 runs were BCLC stage; TNM stage; CLIP stage; cirrhosis status; and gene signatures including Recurrence_Wang (Wang et al., 2007), OS_Lee (Lee et al., 2004), CK19_Andersen (Andersen et al., 2010), Monocyte_Sakai (Sakai et al., 2008), CC_Woo (Woo et al., 2010), MET_Kaposi-Novak (Kaposi-Novak et al., 2006), and EPCAM_Yamashita (Yamashita et al., 2008). For GSE54236, the variables in the random survival forests with positive VIMP values were MET_Kaposi-Novak (Kaposi-Novak et al., 2006), G3_Boyault (Boyault et al., 2007), Proli_Chiang (Chiang et al., 2008), doubling time, CSC_Yamashita (Yamashita et al., 2008), CK19_Andersen (Andersen et al., 2010), CC_Woo (Woo et al., 2010), and TGF-β_Coulouarn (Coulouarn et al., 2008). These results suggest that gene signatures have higher predictive values than conventional factors in predicting of the OS of HCC patient.

### 3.3 Construction of the Functional PPI Network

**Fig. 5:**
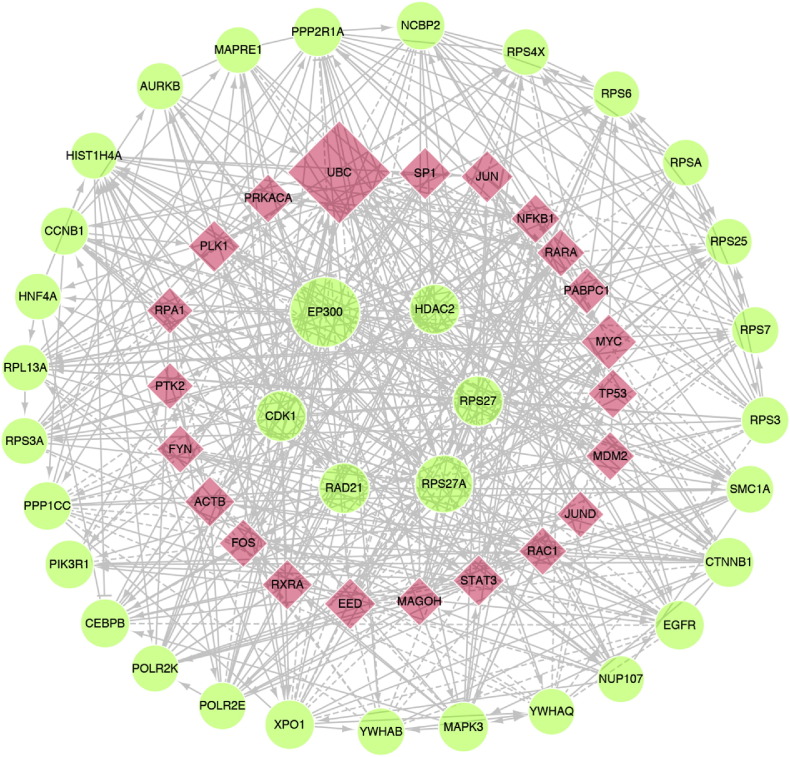
The core functional PPI network derived from the 11 gene signatures that were significantly associated with the overall survival of HCC patients (genes with node degree > 60). The red nodes are genes included in the gene signature, and degree nodes are linker genes in the network construction derived from the Reactome FI plugin of Cytoscape. The size of the node correlates with the degree of the indicated gene in the network. CDK1, HDAC2, RAD21, EP300, RPS27A and RPS27 were genes with the top-ranked degrees.

For the 11 gene signatures that were significantly associated with the OS of HCC patients, 1626 individual genes were covered. With the ReactomeFI plugin in the Cytoscape, a PPI network was constructed with 1406 nodes and 10,135 edges (Fig. S3a). The topological analysis of the PPI network suggested that these 11 gene signatures constituted a scale-free biological signaling network (Fig. S3b-3c). The top-ranked non-linker nodes included EP300, RPS27A, CDK1, RAD21, RPS27, HDAC2, and CTNNB1; the top-ranked linker nodes were UBC, MYC, JUN, SP1, PLK1, EED, ACTB, RXRA, and STAT3 (degree > 90). A simplified PPI network with those genes of node degree > 60 is provided in Fig. 5. We hypothesized that the core genes in the networks may have prognostic predictive values for HCC patients. Because transcription coactivator EP300 and ribosomal proteins RPS27A and RPS27 are not ideal biomarkers or therapeutic targets for HCC, CDK1, RAD21, and HDAC2 were chosen for subsequent analysis because they had relatively high degrees in the PPI network.

### 3.4 Expression Patterns of Potential Prognostic Biomarkers in HCC Tissues

**Fig. 6:**
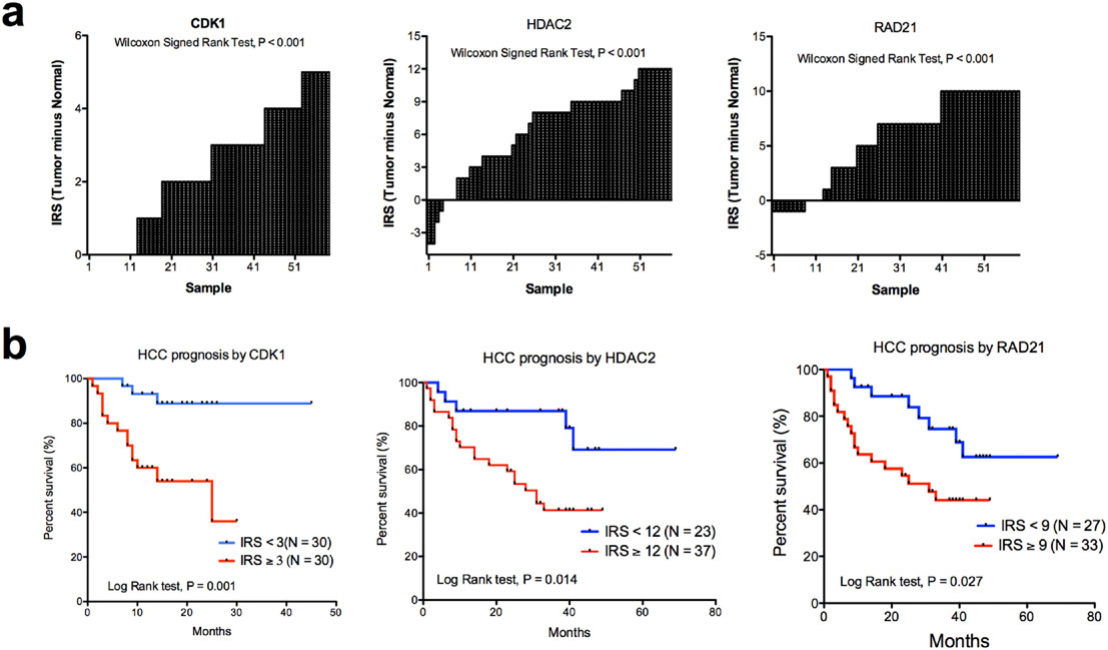
The protein expression levels of RAD21, CDK1, and HDAC2 in tumor and adjacent normal HCC tissues and their associations with overall survival (OS) for HCC patients. (a)CDK1, HDAC2, and RAD21 were significantly increased in HCC tissues compared with adjacent normal tissues (Wilcoxon signed rank test, P < 0.001). (b) Higher expression levels of CDK1 (log-rank test, P = 0.001), HDAC2 (log-rank test, P = 0.014), and RAD21 (log-rank test, P = 0.027) in the HCC tissues were associated with worse OS of the patients compared with lower expression levels.

We constructed TMAs for HCC patients to determine the prognostic values of the core genes with the IHC staining method (N = 60). Detailed patient clinical information is provided in Table S3. We found that the expression levels of CDK1, HDAC2 and RAD21 were increased in tumor tissues compared with the adjacent normal tissues (Fig. 6a). CDK1 was expressed in the cytoplasm of the cancer cells, but no expression was evident in normal tissues (Fig. S4; Wilcoxon test, P < 0.001). Higher expression of CDK1 was associated with worse outcomes for HCC patients (log-rank test, P = 0.001; Fig. 6b). HDAC2 was expressed in the cytoplasm of tumor cells and was overexpressed in HCC tissues (Fig. S4; Wilcoxon test, P < 0.001). Higher expression correlated with poorer OS of HCC patients (log-rank test, P = 0.001; Fig. 6b). RAD21 was immunoreactive mainly in the nuclei of HCC cells (Fig. S4); only weak nuclear staining of RAD21 was evident in normal liver cells (Wilcoxon test, P < 0.001). Higher nuclear staining of RAD21 in HCC cells correlated with worse OS of HCC patients (log-rank test, P = 0.026; Fig. 6b). The univariate analyses of the clinical characteristics and the IHC scores of CDK1, HDAC2, and RAD21 are provided in Table 2. Multivariate Cox analyses showed that CDK1 level, tumor size, and satellite nodule status were independent prognostic factors for the OS of HCC patients (Table 2).

**Table 2:** Univariate and multivariate analysis of the clinical and pathological characteristics for the overall survival of HCC patients (N = 60).

Characteristics	Univariate analysis		Multivariate analysis	
	HR (95% CI)	P-value	HR (95% CI)	P-value
Age, per year	0.98 (0.94–1.02)	0.258		
Sex (Male vs. Female)	0.77 (0.29–2.04)	0.599		
HbeAg (Positive vs. Negative)	0.84 (0.32–2.23)	0.727		
Tumor diameter (> 3 vs. ≤ 3 cm)	3.73 (1.12–12.45)	0.033	3.62 (1.02–12.79)	0.046
Multiple nodules (Yes. vs. No)	5.06 (2.14–7.79)	< 0.001	5.48 (2.09–14.37)	< 0.001
Tumor encapsulation				
Complete vs. Absence	0.58 (0.23–1.47)	0.25		
Incomplete vs. Absence	0.53 (0.20–1.38)	0.192		
Cirrhosis (Child-Pugh B + C vs. A)	1.25 (0.57–2.74)	0.571		
Tumor differentiation (III vs. II)	2.98 (0.70–12.64)	0.138		
Microscopic vascular invasion (Yes vs. No)	3.27 (1.47–7.24)	0.004	2.12 (0.94–4.75)	0.069
BCLC stage (B + C vs. 0 + A)	4.56 (2.09–9.93)	< 0.001		
AFP (> 20 vs. ≤ 20 ng/mL)	2.93 (1.01–8.53)	0.048		
γ-GT (> 50 vs. ≤ 50 U/L)	2.87 (0.99–8.35)	0.053		
RAD21 (High vs. Low)	2.49 (1.08–5.76)	0.033		
HDAC2 (High vs. Low)	3.21 (1.21–8.53)	0.019		
CDK1 (High vs. Low)	3.93 (1.65–9.39)	0.002	4.05 (1.63–10.03)	0.003

Abbreviations: AFP, α-fetoprotein; HR, hazard ratio; 95% CI, 95% confidential interval; BCLC, Barcelona Clinic Liver Cancer stage; γ-GT, γ-glutamyl transpeptidase.

We further validated the prognostic values of CDK1 in an independent case cohort (N = 78; Table S3). HCC patients with higher CDK1levels were associated with poor OS compared with those have lower CDK1 level, as suggested by the log-rank test (P = 0.006; Fig. S5). The multivariate Cox analyses also suggested that CDK1 was also an independent risk factor for HCC patients in the validation cohort (Table S4).

### 3.5 CDK1 is a Potential Therapeutic Target for HCC

The specificity of the antibodies for the IHC staining and the roles of these proteins in HCC progression were confirmed with siRNA methods in cell models. Knockdown of CDK1, RAD21, and HDAC2 with siRNAs led to reduced proliferation of CSQT-2 cells (Fig. S6a). Knockdown of RAD21 reduced the levels of CDK1, but not HDAC2. Knockdown of HDAC2 did not affect the protein expression of CDK1 or RAD21 (Fig. S6b). These results indicate that RAD21 might regulate the CDK1 activity in HCC tumor cells.

Because CDK1 was an independent prognostic factor for HCC, we determined whether CDK1 could be a therapeutic target for HCC. An inhibitor (RO-3306) of CDK1 significantly reduced cellular viability (Supplementary Fig. 6c) and proliferation (Supplementary Fig. 6d) for HCC cell lines, including Hep3B, SMMC-7721, Huh-7 and CSQT-2, suggesting that CDK1 could be a target for HCC treatment.

## 4 Discussion

An analysis of the biological features of HCC is necessary for personalized therapy. Various studies have reported molecular classifications and prognostic molecular signatures for HCC based on the gene expression from HCC tissues or adjacent non-tumoral tissues, which have revealed the tumor heterogeneity [15-42]. With the NTP methods, we determined the prognostic effects of the reported gene signatures in tumor tissues and found that the predictive roles of these gene signatures were reproducible between the datasets. With the random forest survival method, we compared the VIMP values for gene signatures and clinical factors and found that the gene signatures were superior to the clinical characteristics of patients, such as tumor size, nodular status, and the cirrhosis status, for GSE14520 and the doubling time for GSE54236, indicating the values of gene signatures in the prognostic prediction of HCC patients. Interestingly, the gene signatures that were associated with the OS of the patients were in high concordance, and the genes from the signatures were functionally linked with each other. These results suggest that the predictive abilities for the gene signatures are reproducible and that they might contribute to personalized prognosis prediction for HCC patients.

Based on 287 HCC patients, Villanueva et al. determined the predictive effects of early recurrence for 18 gene signatures derived from HCC tissues and 4 from adjacent non-tumor tissues (Villanueva et al., 2011). The genomic gene expression profiling was determined for FFPE tumor specimens and adjacent tissues, and the NTP method was applied to identify those patients with a tendency for early recurrence. They found that only the G3_Boyault gene signature (Boyault et al., 2007), derived from HCC tissues, and another gene signature generated in adjacent non-tumor cirrhotic tissues (Hoshida et al., 2008) were significantly associated with early recurrence or overall recurrence for HCC patients. However, the other 17 tumor-derived gene signatures were not associated with recurrence. Our current study examined the associations between the prediction results of the gene signatures and the recurrence of HCC patients in GSE14520 and found that only gene signature OS_Kim (Kim et al., 2012) was associated with recurrence (data not shown). However, we demonstrated that ten of these gene signatures were predictors for the OS of HCC patients in two independent case cohorts, GSE14520 and GSE54236. These results indicate that, these gene signatures have promising predictive effects for the OS of HCC patients but not for the risk of recurrence. Although disease recurrence is a prognostic factor for the death of HCC patients, other clinical factors and treatment methods also contribute to survival. In future studies, gene signatures that confer the recurrence risk and gene signatures derived from the tumor microenvironment that are associated with the prognosis for HCC patients need to be identified.

Although there were few overlapping genes among the 11 gene signatures (data not shown), they formed a tightly linked, scale-free biological PPI network, as suggested by the node degree distribution following a power algorithm (Barabasi and Oltvai, 2004). We hypothesized that the core genes with high degrees in the network might have important biological functions in the signaling transduction and that their expression levels might have prognostic values. With the in-house HCC tissue samples, the prognostic values for these genes were determined. The expression levels of the core genes RAD21, HDAC2, and CDK1 were increased in HCC tissues compared with adjacent non-tumor tissue, and their expression levels were associated with the OS of HCC patients. As determined with multiple Cox analyses, CDK1, but not RAD21 or HDAC2, when adjusted for clinical characteristics, was an independent prognostic factor for HCC patients.

For various cancer types, HDAC2 acts as an oncogene through the epigenetic regulation of genes and the corresponding signaling cascades in cancer development, and HDAC2 expression is gradually increases, from pre-neoplastic lesions, to low-grade dysplastic nodules, high-grade dysplastic nodules, and HCCs (Nam et al., 2005). Higher HDAC2 levels are correlated with poor survival of HCC patients (Ler et al., 2015, Quint et al., 2011), which is consistent with results of the current study. In HCC cells, inhibiting of HDAC2 disrupts the G1/S phase of the cell cycle and leads to apoptosis through upregulating the total p21, p27, and acetylated p53 levels and reducing CDK6 and BCL2 levels (Lee et al., 2014, Noh et al., 2011); these results suggest that HDAC2 could be a therapeutic target for HCC. In a murine xenograft model, systemic delivery of HDAC2 siRNA encapsulated in lipid nanoparticles reduced the growth of human HCC (Lee et al., 2014); however, this method is far from clinical use. Two small molecule inhibitors of HDACs (HDACis), SAHA (vorinostat) and FK-228 (romidepsin), have been approved by the U.S. Food and Drug Administration (FDA) to treat refractory cutaneous and peripheral T cell lymphoma (West and Johnstone, 2014). In addition to these agents, > 20 different HDACis have produced encouraging results for the treatment of hematological malignancies, including Hodgkin's lymphoma, multiple myeloma, and acute myelocytic leukemia; however, the therapeutic effects of HDACis on solid tumors have been disappointing (West and Johnstone, 2014). In addition to toxicity, the off-target actions of the HDACis may lead to treatment failure for solid tumors. Thus, inhibitors that are selective for HDAC2 should be developed, and their therapeutic effects in HCC patients need to be addressed.

RAD21, a component of the cohesion complex, is essential for chromosome segregation during the metaphase-anaphase transition of mitosis (Xu et al., 2004). RAD21 is also involved in homologous recombinational repair or the error-free repair of DNA damage, which could influence the sensitivity of gastrointestinal and breast cancers to radiotherapy or chemotherapy (Xu et al., 2010, Xu et al., 2011). High levels of nuclear RAD21 staining correlate with poor disease-specific survival of colorectal cancer patients with KRAS mutations (Deb et al., 2014) and with early relapse in patients with high-grade luminal, basal, or HER2 breast cancers (Xu et al., 2011). However, the roles of RAD21 in HCC development and progression have not been determined. In the current study, we found that nuclear RAD21 was increased in HCC tissues compared with adjacent non-tumor tissues, and higher RAD21 levels were associated with shorter OS of HCC patients. In HCC cells, RAD21 knockdown reduced cellular proliferation and down-regulated CDK1 levels, which might partially underlie the RAD21 oncogenic activities in HCCs. These results suggest that RAD21 could be a biomarker or therapeutic target for HCC patients and that the potential intervention methods need to be developed.

CDK1 is a cyclin-dependent kinase that plays critical roles in the regulating of cellular mitosis. Depending on its association with cyclin A or B, it participates in the progression of the G1/S and G2/M phases of the cell cycle through the phosphorylation of various substrates, including Ajuba (Chen et al., 2016), CDP/Cux (Santaguida et al., 2001), Bcl2 (Vantieghem et al., 2002), and Wee1 (Harvey et al., 2005) (Petrone et al., 2016). Quantitative phosphoproteomics has identified > 500 candidate substrates for CDK1; these substrates are associated with genes that are G2 and M phase-specific (Petrone et al., 2016). In addition to its roles in mitosis, CDK1 also participates in the regulation of self-renewal, differentiation, and somatic reprogramming of human embryonic stem cells (Wang et al., 2017). CDK1 also stimulates the enzymatic activity of SIRT3, which enhances mitochondrial function and tumor radioresistance (Liu et al., 2015). Hyperactivation of CDK1 is associated with poor prognosis for patients with lung adenocarcinoma (Shi et al., 2016), ovarian cancer (Yang et al., 2016), renal cell carcinoma(Hongo et al., 2014), and breast cancer (Pavlou et al., 2014). For lymphomas, hepatoblastomas, and breast cancers, the inhibition of CDK1 downregulates survivin expression and induces MYC-dependent apoptosis (Goga et al., 2007, Kang et al., 2014). In the current study, we found that CDK1 was detectable in HCC cells but not in normal liver tissues and that high CDK1 independently correlated with short OS. Considering its biological roles, CDK1 expression in HCC cells might reflect proliferation status and cancer stem cell properties, which are associated with the OS of HCC patients (Fig. 1b and d). As determined with cultured cells, CDK1 knockdown or inhibition correlated with reduced cellular proliferation, suggesting that CDK1 is a therapeutic target for HCC. Various inhibitors of CDK1, including flavopiridol, BMI-1026, olomoucine, staurosporine, and RO-3306 have been developed, and some have entered phase I and II clinical trials for the treatment of a variety of solid tumors and hematologic malignancies (Wang et al., 2011). For most of these inhibitors, however, their selective activity is poor; they generally inhibit CDK1, 2, 4, and 6 with equal potency (Wang et al., 2011). As shown in early clinical trials, these inhibitors often lead to high toxicity. Selective CDK1 inhibitors can exert more favorable therapeutic effects. For example, a selective CDK1 inhibitor, RO-3306, induces cell cycle arrest and apoptosis in cancer cells but has minimal effects on normal cells (Vassilev et al., 2006). In the current study, RO-3306 reduced the proliferation of cultured HCC cells. However, whether selective inhibitors for CDK1 could act as monotherapy agents or show synergistic effects with other chemotherapeutic agents needs to be addressed.

In conclusion, we evaluated the prognostic capacity of 30 gene signatures and found that 11 were significantly associated with the OS of HCC patients in two HCC cohorts. The genes from the 11 signatures consisted of a scale-free functional PPI network. The protein expression levels of the core nodes, RAD21, CDK1, and HDAC2, had prognostic value for HCC patients; however, only CDK1 was an independent prognostic factor. Further, because inhibition of CDK1 showed promising anticancer activity, it could serve as a therapeutic target for HCCs. In summary, the results provide potential biomarkers for the prediction of prognosis and present potential targets for the treatment of HCC patients. However, more studies are warranted to determine the roles of these proteins and to develop novel therapeutics for HCC.

## Funding

The work was financially supported by the by grants from the Ministry of Science and Technology of the People's Republic of China (2014AA020524), the Special Research Fund of Ministry of Health for Non-Profit Sector (201302010), the National Nature Science Foundation (81302507), the Science and Technology Commission of Shanghai Municipality (14391901800), and the Shanghai Municipal Commission of Health and Family Planning (20164Y0250, 2015ZB0202). The funders had no role in study design, data collection and analysis, decision to publish, or preparation of the manuscript.

## Conflict of Interest

The authors have declared that no competing interests exist.

## Author Contributions

P.C., X.Z. and J.Z. contributed to the study concept and design; P.C. J.Z. and X.Z. obtained funding and provided the essential materials; J.C., B.L., Y.Z., X.F. M.Z. and S.L. performed the experiments; X.J., J.Z. and J.W. analyzed the data; P.C. and X.Z. wrote the manuscript. All authors reviewed and approved the final manuscript.

## Appendix A Supplementary data

Supplementary Information Image 1
