# Supplementary material for: Prognostic Biomarker Identification Through Integrating the Gene Signatures of Hepatocellular Carcinoma Properties
Source: eBioMedicine. 2017 Apr 12;19:18–30. doi: 10.1016/j.ebiom.2017.04.014 (PMC5440601; doi:10.1016/j.ebiom.2017.04.014)

Supplementary Tables and Figures

Table S1. Univariate Cox regression analyses for the influences of the clinical characteristics on the overall survival of HCC patients in datasets GSE14520 and GSE54236.

Table S2. Univariate Cox regression analyses of the nearest template prediction (NTP) results of the gene signatures and the overall survival of HCC patients in datasets GSE14520 (N = 242) and GSE54236 (N = 78).

Table S3. Eleven gene signatures that were associated with the overall survival of HCC patients in the two independent cohorts.

Table S4. The basic characteristics of the HCC patients in the tissue microarray construction and IHC tests.

Table S5. Univariate and multivariate analyses of the clinical and pathological characteristics for the OS of the HCC patients in validation cohort (N = 78).

Fig. S1. Kaplan-Meier plots and log-rank tests for the 8 gene signatures that were not associated with the overall survival of HCC patients in GSE14520.

Fig. S2. Kaplan-Meier plots and log-rank tests for the 11 gene signatures that were not associated with the OS of HCC patients in GSE54236.

Fig. S3. The functional PPI network derived from the 11 gene signatures that were significantly associated with the overall survival of the HCC patients in datasets GSE14520 (N = 242) and GSE54236 (N = 78). (a) An organic layout for the PPI network of the gene signatures. Green nodes are genes that are included in the gene signatures; red nodes are those linker genes inferred from the Reactome FI plugin of

Cytoscape. (b) Nodes degree distribution corresponding to their degree. A power law was fitted by $Y = 423.2*X^{-1.224}$ ($R^2 = 0.865$). The x- and y-axes are presented as logarithm scales. (c) Clustering coefficient distribution of the nodes; the x-axis is presented as a logarithm scale. (d) Path length distribution between the nodes.

Fig. S4. Representative figures for protein expression patterns in HCC (low, moderate or high) and adjacent normal tissues.

Fig. S5. The Kaplan-Meier plot and log-rank test for the overall survival of HCC patients in the validation group (N = 78) with higher vs. lower expression of CDK1.

Fig. S6. CDK1 inhibition reduces the viability and proliferation of HCC cells. (a) The OD450 values for cellular proliferation of CSQT-2 cells with RAD21, CDK1, or HDAC2 siRNA knockdown and the corresponding control group. **P < 0.001 for one-way ANONA tests among the groups. (b) Western blotting for the endogenous proteins in the RAD21, CDK1, or HDAC2 siRNA-transfected CSQT-2 cells. (c) Dose-responses of RO-3306 on the viability of CSQT-2, Huh-7, Hep3B and SMMC-7721 cells. (d) RO-3306 (12.5 μM) significantly inhibited the cellular proliferation of CSQT-2, Hep3B and Huh-7 cells. **P < 0.01 for the control group vs. the RO-3306-treated group for each cell line at the indicated time (Student's t-tests).

Table S1. Univariate Cox regression analyses for the influences of the clinical characteristics on the overall survival of HCC patients in datasets GSE14520 and GSE54236.

Variable	GSE14520 dataset				GSE54236 dataset			
	N	HR	95% CI	P	N	HR	95% CI	P
Gender (Male vs. Female)	242	1.86	0.90-3.83	0.093	78	1.23	0.69-2.19	0.486
Age (>50 year vs. < 50 years)	242	0.92	0.62-1.38	0.688				
HBV (CC vs AVR_CC)	224							
CC vs. AVR_CC		0.74	0.46-1.17	0.196				
N vs. AVR_CC		0.71	0.17-2.99	0.638				
Tumor size (Large vs. small)	241	1.96	1.31-2.93	0.001				
Nodular number (Multiple vs. Single)	242	1.65	1.06-2.57	0.025				
Cirrhosis (Yes vs. No)	242	5.09	1.26-20.6 8	0.023				
BCLC	225							
B vs. A+0		2.67	1.47-4.85	0.001				
C vs. A+0		4.97	2.97-8.34	< 0.001				
CLIP	225							
I vs. 0		1.48	0.87-2.52	0.152				
II vs. 0		3.26	1.84-5.76	< 0.001				
III vs. 0		6.58	3.15-13.7 3	< 0.001				
AFP (High vs. Low)	238	1.69	1.13-2.53	0.011				
ALT (high vs. Low)	242	0.87	0.58-1.30	0.483				
TNM	225							
II vs. I		2.16	1.25-3.75	0.006				
III vs. I		5.41	3.11-9.41	< 0.001				
Doubling Time (Low vs. High)					78	0.50	0.30-0.83	0.007

Table S2. Univariate Cox regression analyses of the nearest template prediction (NTP) results of the gene signatures and the overall survival of HCC patients in datasets GSE14520 (N = 242) and GSE54236 (N = 78).

Variable	GSE14520 dataset				GSE54236 dataset			
	N	HR	95% CI	P	N	HR	95% CI	P
CC_Woo	242				78			
Unclassified vs. Good		1.35	0.73-2.49	0.342		1.71	0.87-3.35	0.12
Poor vs. Good		2.55	1.65-3.95	< 0.001		1.79	1.08-2.95	0.023
OS_Lee	242				78			
Unclassified vs. Good		1.66	0.91-3.04	0.099		0.41	0.18-0.94	0.035
Poor vs. Good		3.13	1.99-4.91	< 0.001		1.75	1.08-2.86	0.025
Proliferation_Chiang	242				78			
Unclassified vs. Good		1.05	0.56-1.96	0.876		0.92	0.46-1.85	0.817
Poor vs. Good		2.42	1.56-3.76	< 0.001		2.36	1.43-3.92	< 0.001
G3_Boyault	242				78			
Unclassified vs. Good		2.02	1.16-3.51	0.012		0.97	0.52-1.83	0.934
Poor vs. Good		2.34	1.46-3.75	< 0.001		2.63	1.58-4.39	< 0.001
S2_Hoshida	242				78			
Unclassified vs. Good		1.46	0.88-2.43	0.142		1.69	1.02-2.80	0.042
Poor vs. Good		1.81	1.13-2.89	0.014		2.51	1.26-4.99	0.009
CK19_Andersen	242				78			
Unclassified vs. Good		1.08	0.63-1.85	0.785		1.18	0.67-2.09	0.571
Poor vs. Good		2.62	1.65-4.15	< 0.001		2.69	1.55-4.65	< 0.001
Recurrence_Woo	242				78			
Unclassified vs. Good		1.86	1.13-3.05	0.014		0.96	0.53-1.17	0.898
Poor vs. Good		2.26	1.39-3.68	0.001		1.13	0.68-1.89	0.636
OS_Kim	242				78			
Unclassified vs. Good		1.64	1.01-2.65	0.048		1.14	0.66-1.99	0.636
Poor vs. Good		2.08	1.28-3.40	0.003		1.27	0.74-2.19	0.388
CSC_Yamashita	242				78			
Unclassified vs. Good		1.62	1.03-2.56	0.037		1.63	1.01-2.65	0.046
Poor vs. Good		2.09	1.19-3.65	0.01		3.16	1.41-7.08	0.005
EPCAM_Yamashita	242				78			
Unclassified vs. Good		1.99	1.09-3.63	0.026		1.28	0.73-2.25	0.383
Poor vs. Good		3.19	1.73-5.88	< 0.001		2.79	1.37-5.68	0.005
VI_Minguez	242				78			
Unclassified vs. Good		1.42	0.87-2.31	0.165		1.42	0.88-2.29	0.146
Poor vs. Good		2.62	1.45-4.74	0.001		3.64	1.25-10.58	0.018
C2_Cario	242				78			
Unclassified vs. Good		1.66	1.01-2.73	0.047		0.85	0.51-1.45	0.558
Poor vs. Good		3.05	1.61-5.80	< 0.001		2.1	0.92-4.80	0.079
Recurrence_Wang	242				78			
Unclassified vs. Good		2.11	1.12-4.00	0.021		1.69	0.93-3.06	0.084
Poor vs. Good		2.89	1.35-6.16	0.006		3.16	1.17-8.50	0.023

Table S2. Continued.

Variable	GSE14520 dataset				GSE54236 dataset			
	N	HR	95% CI	P	N	HR	95% CI	P
MET_Kaposi-Novak	242				78			
Unclassified vs. Good		2.77	1.28-6.01	0.01		0.98	0.51-1.88	0.947
Poor vs. Good		2.86	1.13-7.28	0.027		6.04	2.58-14.12	< 0.001
Monocyte_Sakai	242				78			
Unclassified vs. Good		0.6	0.38-0.96	0.034		1.16	0.59-2.29	0.662
Poor vs. Good		5.9	1.98-17.58	0.001		1.43	0.62-3.03	0.402
S1_Hoshida	242				78			
Unclassified vs. Good		1.69	1.04-2.72	0.033		1.61	0.91-2.86	0.103
Poor vs. Good		1.52	0.92-2.50	0.102		1.44	0.85-2.41	0.173
CTNNB1_Chiang	242				78			
Unclassified vs. Good		1.37	0.87-2.17	0.176		1.65	0.94-2.91	0.084
Poor vs. Good		0.8	0.44-1.47	0.478		1.49	0.84-2.63	0.169
TGFB_Coulouarn	242				78			
Unclassified vs. Good		1.21	0.75-1.97	0.438		1.1	0.67-1.81	0.715
Poor vs. Good		1.77	0.98-3.18	0.056		2.23	1.17-4.28	0.016
Interfron_Chiang	242				78			
Unclassified vs. Good		1.43	0.79-2.58	0.243		1.28	0.68-2.39	0.443
Poor vs. Good		0.89	0.43-1.84	0.747		0.66	0.32-1.36	0.263
G5/6_Boyault	242				78			
Unclassified vs. Good		0.71	0.43-1.16	0.173		1.18	0.60-2.32	0.639
Poor vs. Good		0.57	0.28-1.13	0.11		1.73	0.73-4.13	0.214
VAG_Ko	242				78			
Unclassified vs. Good		1.64	0.75-3.56	0.215		0.7	0.33-1.47	0.344
Poor vs. Good		1.74	0.74-4.09	0.206		1.12	0.45-2.80	0.814
G6_Boyault	242				78			
Unclassified vs. Good		1.06	0.59-1.92	0.849		1.16	0.62-2.18	0.646
Poor vs. Good		0.76	0.35-1.65	0.491		1.52	0.69-3.32	0.300
G2_Roessler	242				NA			
Unclassified vs. Good		2.56	1.03-6.30	0.042				
Poor vs. Good		2.93	0.85-10.12	0.090				
Lymphocyte_Chew	NA				78			
Unclassified vs. Good						1.59	0.92-2.75	0.094
Poor vs. Good						1.47	0.49-4.38	0.49

Abbreviations: HR, hazard ratio; 95% CI, 95% confidential interval; NA, not applicable.

Table S3. Eleven gene signatures that were associated with the overall survival of HCC patients in the two independent cohorts.

**No. of the gene signature**	**Gene signature**
1	CK19_Andersen
2	S2_Hoshida
3	CC_Woo
4	VI_Minguez
5	Recurrence_Wang
6	CSC_Yamashita
7	EPCAM_Yamashita
8	OS_Lee
9	MET_Kaposi-Novak
10	Proliferation_Chiang
11	G3_Boyault

Table S4. The basic characteristics of the HCC patients in the tissue microarray construction and IHC tests.

**Characteristics**	**HCC cohort I (N = 60)**	**Validation Cohort (N = 78)**	**P**
Age (year, ±SD)	51.8±9.67	49.2±10.23	< 0.001
Sex (Male/Female)	50/10	73/5	0.100
HbeAg (Positive vs. Negative)	15/45	22/56	0.820
Tumor diameter (> 3 vs. ≤3 cm)	44/16	59/19	0.911
Multiple nodes (No. vs. Yes)	52/8	44/34	< 0.001
Tumor encapsulation (Complete/Incomplete/Absence)	23/23/14	32/21/25	0.308
Cirrhosis (Child-Pugh B+C vs. A)	24/36	14/64	0.008
Tumor differentiation (III vs. II+I)	50/10	60/18	0.475
Microscopic vascular invasion (Yes vs. No)	13/47	36/42	0.005
BCLC stage (B+C vs. 0+A)	18/42	38/40	0.041
AFP (> 20 vs. ≤ 20 ng/mL)	42/18	55/23	1.000
γ-GT (> 50 vs. ≤50 U/L)	43/17	NA	NA

Abbreviations: AFP, α-fetoprotein; BCLC, Barcelona Clinic Liver Cancer stage; γ-GT, γ-glutamyl transpeptidase; NA, not applicable.

Table S5. Univariate and multivariate analyses of the clinical and pathological characteristics for the OS of the HCC patients in validation cohort (N = 78).

**Characteristics**	**Univariate analysis**		**Multivariate analysis**	
	**HR (95% CI)**	**P-value**	**HR (95% CI)**	**P-value**
Age, per year	1.01 (0.97-1.05)	0.811		
Sex (Male vs. Female)	1.55 (0.21-11.47)	0.671		
HbeAg (Positive vs. Negative)	0.47 (0.16-1.38)	0.167		
Tumor diameter (> 3 vs. ≤3 cm)	1.55 (0.53-4.57)	0.424		
Multiple nodule (Yes vs. No)	4.25 (1.67-10.78)	0.002	3.86 (1.52-9.83)	0.005
Tumor encapsulation				
Complete vs. Absence	0.28 (0.10-0.80)	0.017		
Incomplete vs. Absence	0.52 (0.20-1.40)	0.198		
Cirrhosis (Child-Pugh B+C vs. A)	1.12 (0.41-3.01)	0.828		
Tumor differentiation (III vs. II)	2.19 (0.65-7.38)	0.205		
Microscopic vascular invasion (Yes vs. No)	2.69 (1.14-6.34)	0.024		
BCLC stage (B+C vs. 0+A)	2.87 (1.18-6.99)	0.020		
AFP (> 20 vs. ≤ 20 ng/mL)	2.28 (0.78-6.72)	0.133		
CDK1 (High vs. Low)	3.14 (1.33-7.41)	0.009	2.78 (1.18-6.59)	0.020

Abbreviations: AFP, α-fetoprotein; HR, hazard ratio; 95% CI, 95% confidential interval; BCLC, Barcelona Clinic Liver Cancer stage.

Fig. S1. Kaplan-Meier plots and log-rank tests for the 8 gene signatures that were not associated with the overall survival of HCC patients in GSE14520.

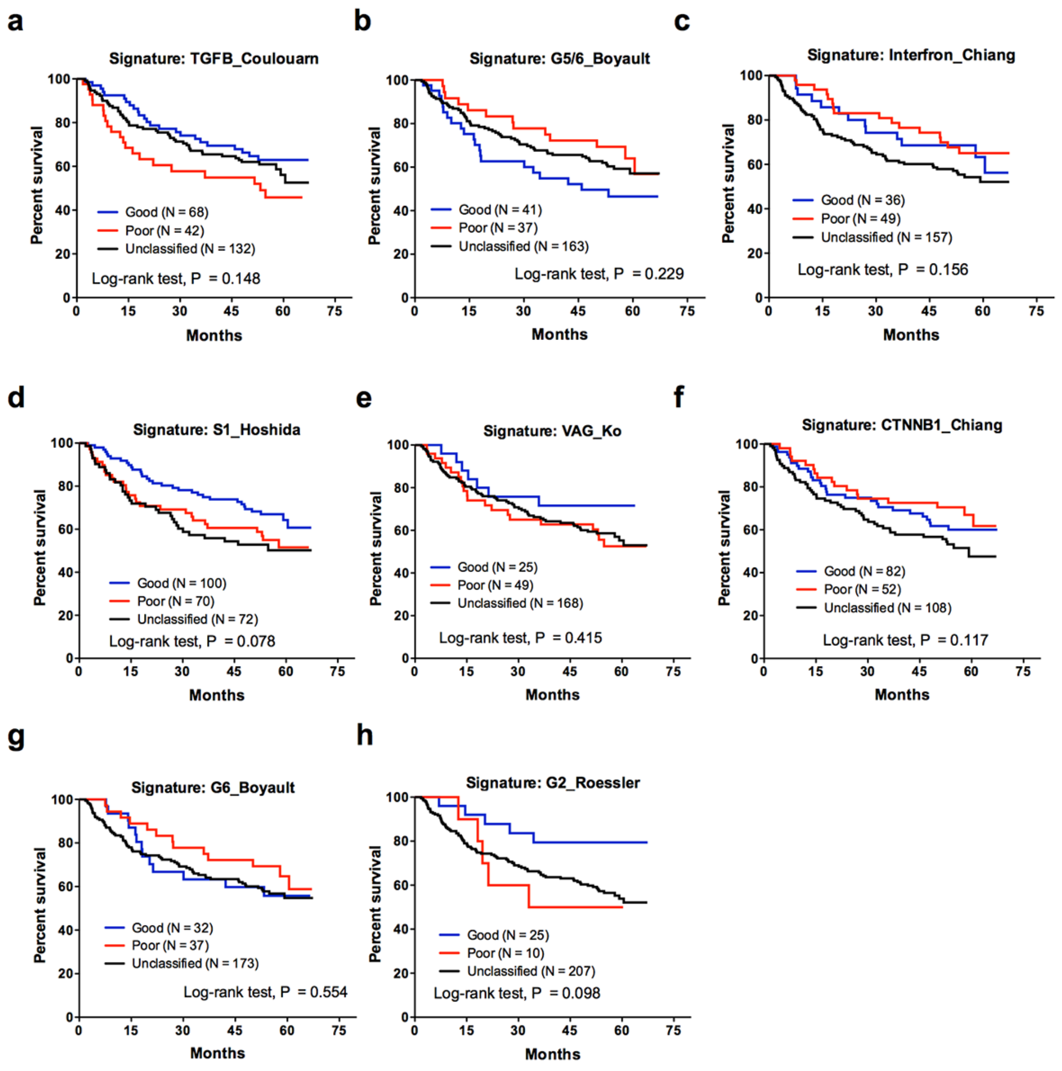

Fig. S2. Kaplan-Meier plots and log-rank tests for the 11 gene signatures that were not associated with the OS of HCC patients in GSE54236.

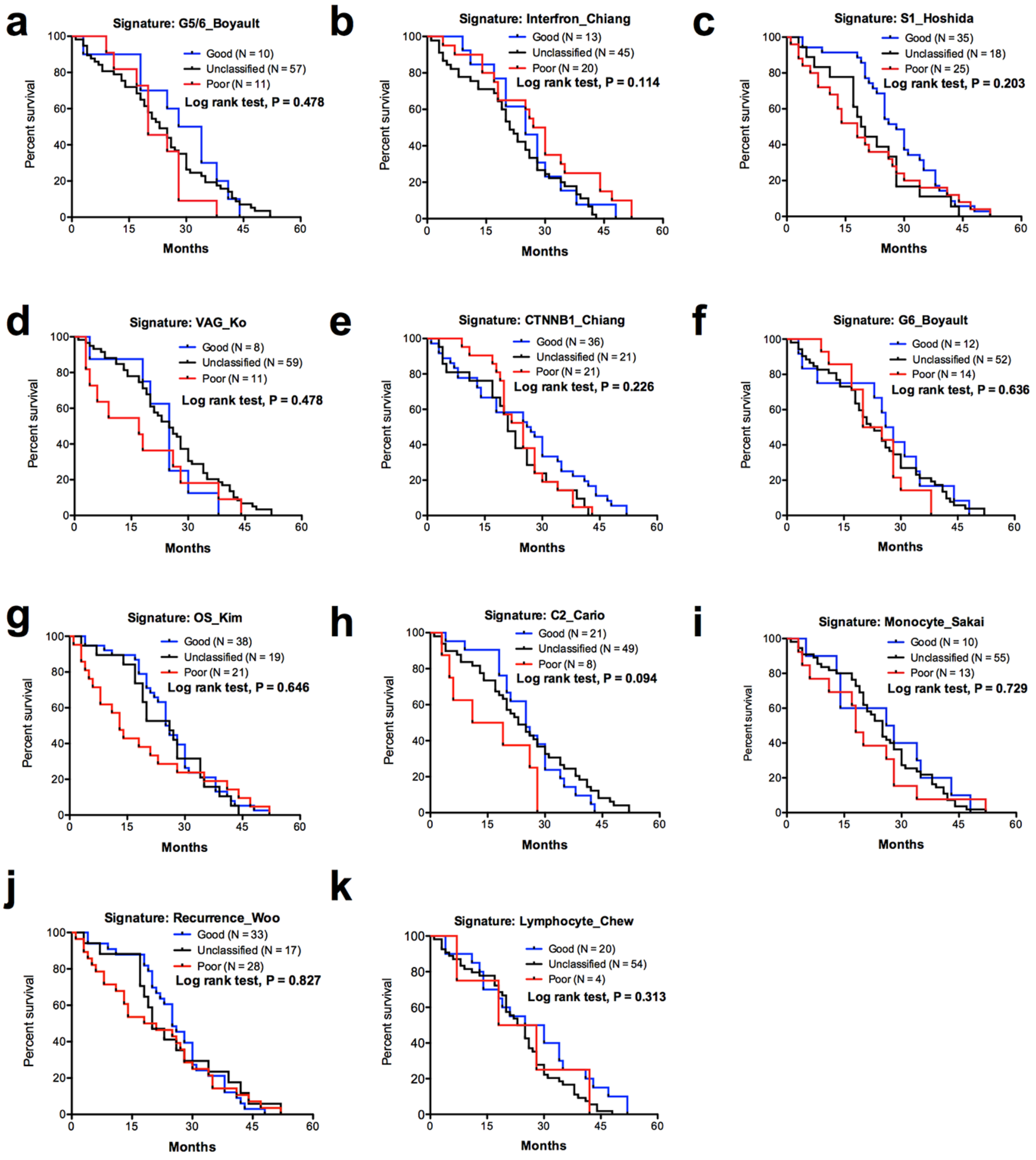

Fig. S3. The functional PPI network derived from the 11 gene signatures that were significantly associated with the overall survival of the HCC patients in datasets GSE14520 (N = 242) and GSE54236 (N = 78). (a) An organic layout for the PPI network of the gene signatures. Green nodes are genes that are included in the gene signatures; red nodes are those linker genes inferred from the Reactome FI plugin of Cytoscape. (b) Nodes degree distribution corresponding to their degree. A power law was fitted by $Y = 423.2*X^{-1.224}$ ($R^2 = 0.865$). The x- and y-axes are presented as logarithm scales. (c) Clustering coefficient distribution of the nodes; the x-axis is presented as a logarithm scale. (d) Path length distribution between the nodes.

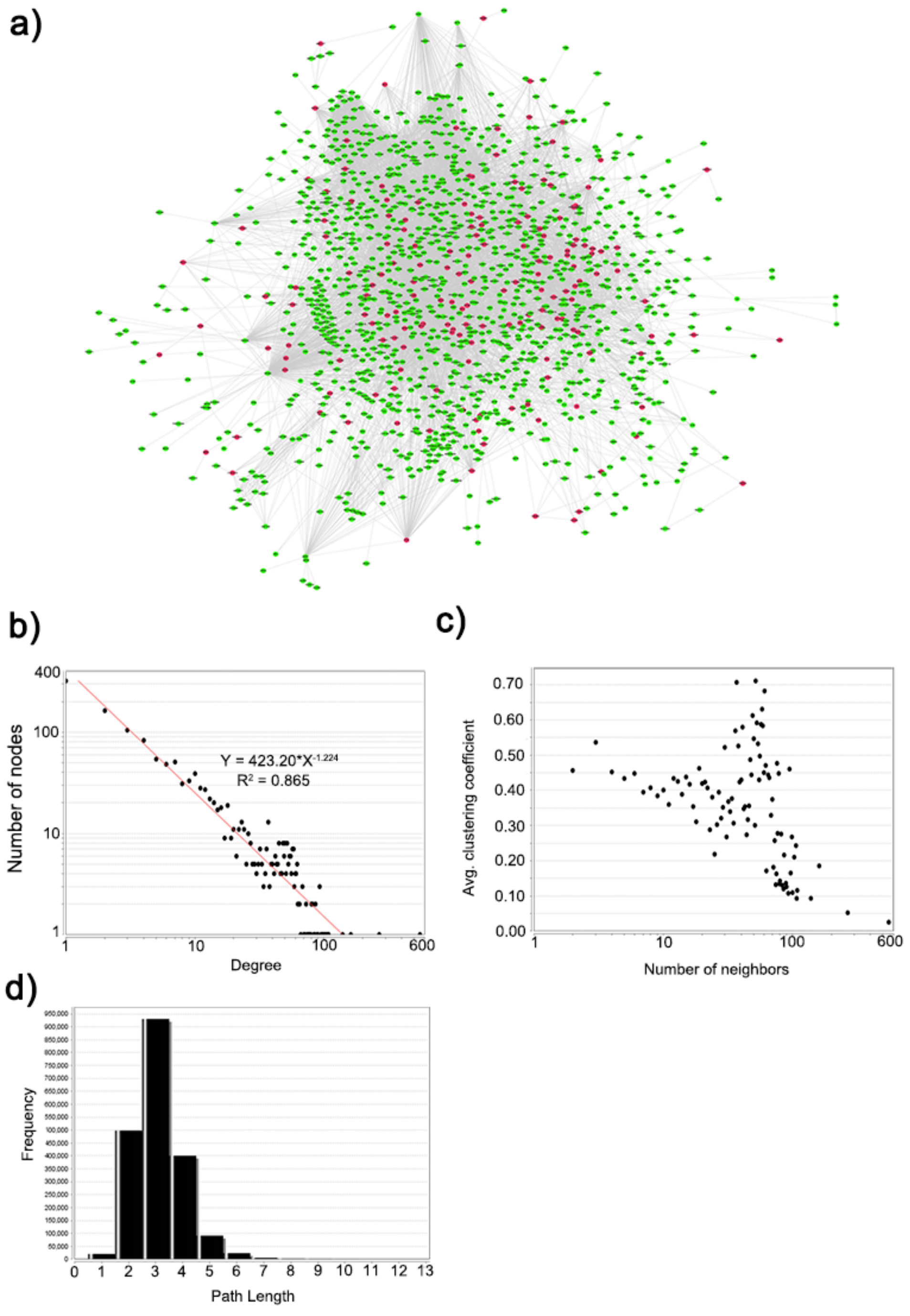

Fig. S4. Representative figures for protein expression patterns in HCC (low, moderate or high) and adjacent normal tissues.

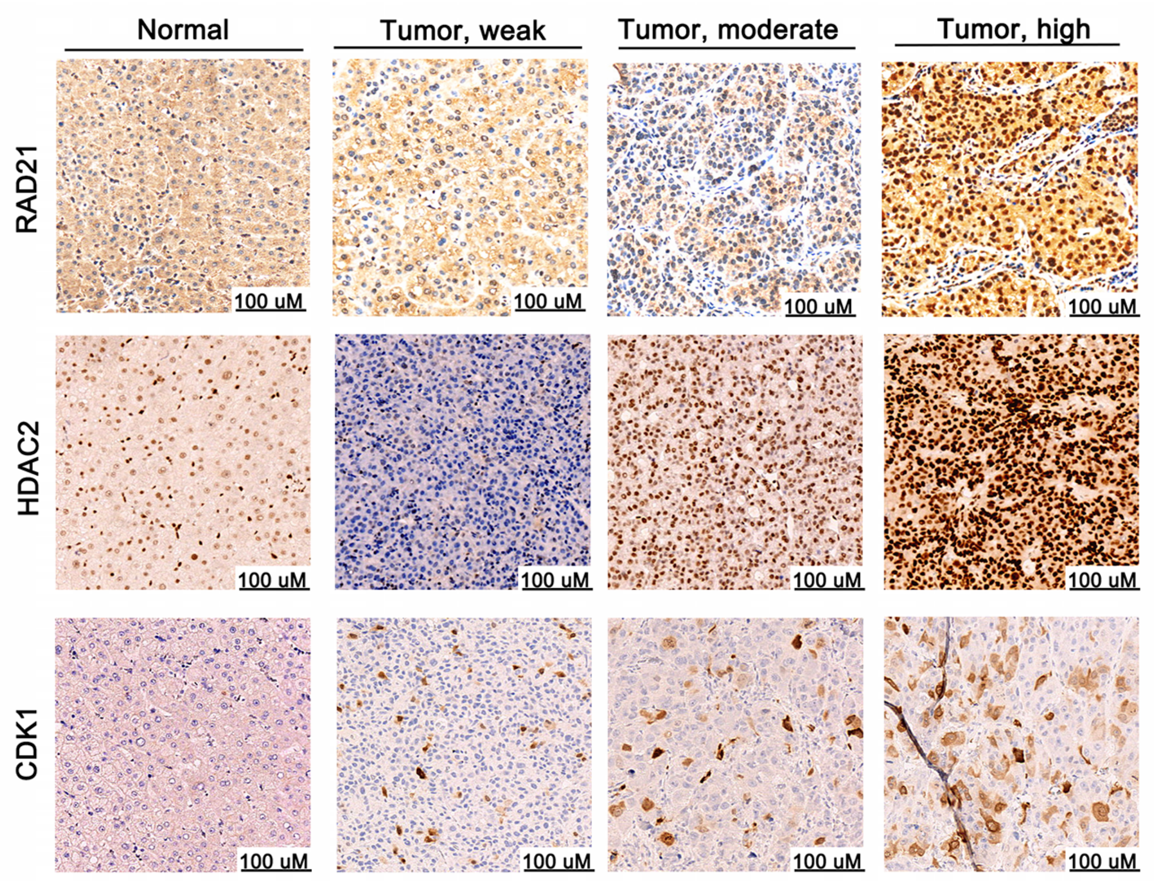

Fig S5. The Kaplan-Meier plot and log-rank test for the overall survival of HCC patients in the validation group (N = 78) with higher vs. lower expression level of CDK1.

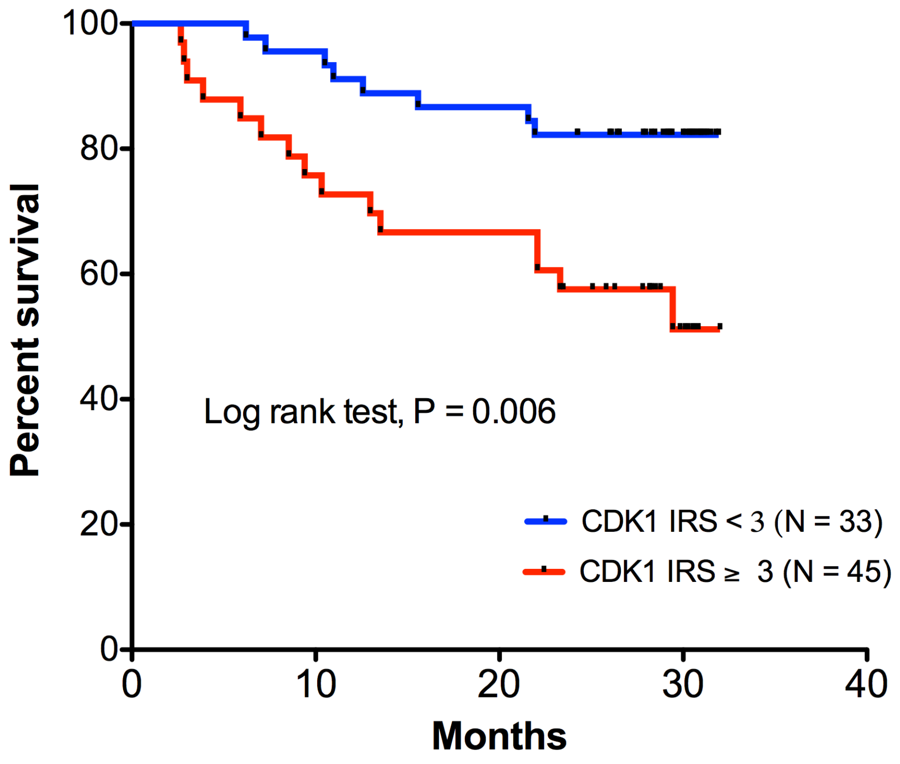

Fig. S6. CDK1 inhibition reduces the viability and proliferation of HCC cells. (a) The OD450 values for cellular proliferation of CSQT-2 cells with RAD21, CDK1, or HDAC2 siRNA knockdown and the corresponding control group. **P < 0.001 for one-way ANONA tests among the groups. (b) Western blotting for the endogenous proteins in the RAD21, CDK1, or HDAC2 siRNA-transfected CSQT-2 cells. (c) Dose-responses of RO-3306 on the viability of CSQT-2, Huh-7, Hep3B and SMMC-7721 cells. (d) RO-3306 (12.5 μM) significantly inhibited the cellular proliferation of CSQT-2, Hep3B and Huh-7 cells. **P < 0.01 for the control group vs. the RO-3306-treated group for each cell line at the indicated time (Student's t-tests).

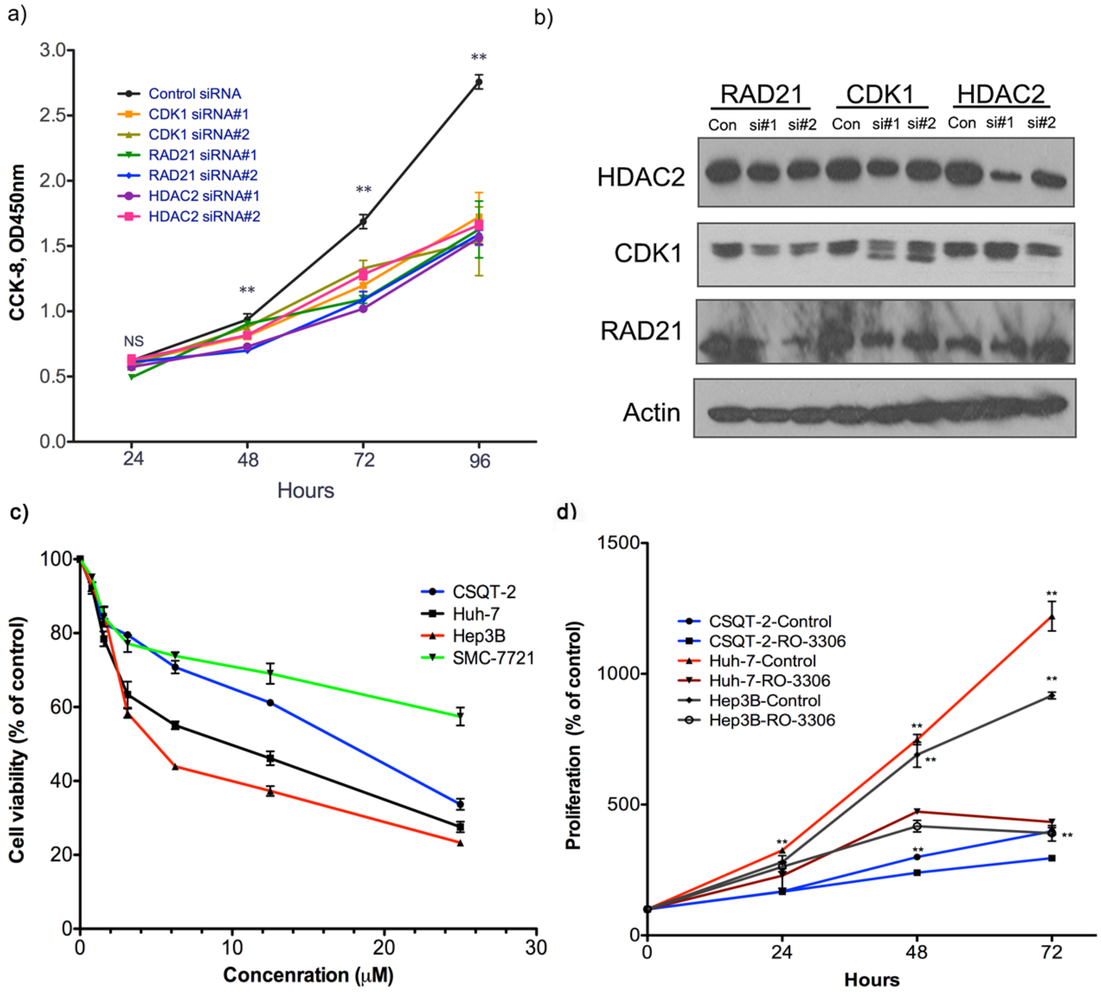

Supplement: Supplementary file 1 — Supplementary Information [file mmc1.pdf]
